# Re-Starting the Cruise Sector during the COVID-19 Pandemic in Greece: Assessing Effectiveness of Port Contingency Planning

**DOI:** 10.3390/ijerph192013262

**Published:** 2022-10-14

**Authors:** Lemonia Anagnostopoulos, Leonidas Kourentis, Antonios Papadakis, Varvara A. Mouchtouri

**Affiliations:** 1 Laboratory of Hygiene and Epidemiology, Faculty of Medicine, University of Thessaly, 22 Papakyriazi Street, 41222 Larisa, Greece; 2 EU Joint Action HEALTHY GATEWAYS, 22 Papakyriazi Street, 41222 Larisa, Greece; 3 Department of Clinical Microbiology and Microbial Pathogenesis, School of Medicine, University of Crete, Voutes–Staurakia, 71110 Heraklion, Greece

**Keywords:** contingency planning, COVID-19, cruise, intra-action review, maritime, point of entry, port, SARS-CoV-2, ship, travel

## Abstract

Coronavirus disease (COVID-19) outbreaks on board cruise ships early in the pandemic highlighted gaps worldwide in public health emergency contingency plans (PHECPs) for responding to unknown threats. To restart cruise operations in 2021 and respond to potential COVID-19 outbreaks, a major tourist-based Greek island port (Port A) developed a COVID-19 PHECP. We assessed plan effectiveness by reviewing epidemiological data and monitoring outcomes, followed by an intra-action review (IAR) analyzing three event responses. From May to December 2021, 118 calls from 23 cruise ships with 119,930 passengers were recorded, with 29 COVID-19 cases in 11 cruises on board 7 ships. No outbreak was recorded during the study period. Strengths of the introduced PHECP included commitment of senior management; a core multi-disciplinary team of local authorities/ship agents involved in design and execution; interoperability agreements for port and ships’ PHECPs; cruise industry commitment to compliance; and pre-existing scenarios considering capacity needs. Central government coordination for preparedness planning at local ports is essential for successful responses. Monitoring local and country level response capacities is critical to inform planning, risk assessment, and decision-making. Immediately recording ports’ response actions provides the basis to capture lessons and improve contingency plans. To facilitate communication and common response protocols between European and non-European ports, IARs should be conducted between countries.

## 1. Introduction

The coronavirus disease (COVID-19) pandemic presented a significant challenge to port authorities, highlighting gaps in ports’ public health preparedness and response capacities as well as existing port plans worldwide [1]. International Health Regulations (IHR) 2005 require that designated ports have capacities to provide appropriate public health emergency responses, including establishment and maintenance of a public health emergency contingency plan (PHECP) for known and unknown public health risks [2]. Other public health capacities that designated ports should have in place, either at all times or for emergency responses, include capacities to transport ill travelers to appropriate facilities, pre-defined arrangements for isolation and care of affected travelers, and provisions for quarantine of suspected travelers [2]. Difficulties reported in responding to COVID-19 outbreaks on passenger ships during the early stages of the pandemic were often related to insufficient public health capacities. Challenges with arranging medical facilities and transport for affected travelers during outbreaks demonstrated the importance of ensuring adequate public health capacities are in place for responding to potential outbreaks from new emerging pathogens on passenger ships [3,4,5,6,7]. Early in the pandemic, outbreaks resulted in varied responses, with some instances reported of cruise ships being refused entry to ports, and refusal of the disembarkation of travelers [4,8]. 

The unprecedented suspension of cruise ship travel, beginning in March 2020, had a substantial economic impact. This resulted in an estimated USD 50 billion loss in economic activity globally in 2020, with over EUR 7 billion in lost wages and 215,800 lost jobs in Europe alone [9]. By May 2020, both governmental authorities and the cruise industry attempted to develop protocols to support the safe restart of cruise operations. With global guidance from the World Health Organization (WHO) [10] and European guidance from the European Union (EU), joint action HEALTHY GATEWAYS [11,12] was developed. Guidance addressed operations onboard cruise ships and at local ports, as well as central level governmental planning, preparedness, and response capacities. HEALTHY GATEWAYS further produced a tool to support development and assessment of port contingency plans, incorporating guidance to prepare COVID-19 specific plans [13]. Recognizing the need for countries to quickly identify lessons learned and subsequently modify their COVID-19 response during the ongoing pandemic, WHO introduced the intra-action review (IAR) framework [14]. This framework is based on the principles of the more well-established after-action review (AAR), a component of the IHR Monitoring and Evaluation Framework [15]. While the AAR systematically reviews response actions following a public health event to improve preparedness and response capacities, the IAR framework has a narrower scope and smaller scale; an IAR can be conducted rapidly by countries during a public health event [14,15]. Through facilitated discussion, an IAR allows stakeholders involved in the response to document good practices, challenges, lessons learned, and identify actions that can be immediately implemented to improve a country’s current public health emergency response [15,16,17]. IARs can be conducted not only at national level, but can be tailored to different levels and settings. Both WHO and the European Centre for Disease Prevention and Control (ECDC) have developed guidance to support countries with conducting IARs in the context of the COVID-19 pandemic [16,17].

Greece relies heavily on travel and tourism activities. Suspension of cruise travel in response to COVID-19 led to significant declines in cruise calls and passengers from 2019 to 2020. Survey data from the Bank of Greece concluded the country saw a 98% reduction in total receipts from cruise passengers during this period [18]. To support cruise travel resumption in 2020 and 2021, the Greek Ministry of Health updated public health protocols to be enforced at Greek ports. These protocols followed guidance published by HEALTHY GATEWAYS [11,12,13], ECDC, and the European Maritime Safety Agency (EMSA) [19]. Protocols addressed essential prerequisites for interoperability of port and cruise ship contingency plans, roles of home/contingency/transit ports in cruise itineraries, and defined a port’s maximum capacity for cruise ships and travelers considering local public health capacities. 

One such tourist-based port (Port A) is located on a Greek island in the eastern Mediterranean Sea supporting cruise, ferry, and cargo operations. Its cruise itineraries commonly include ports of call in both European and non-European countries. Situated in a summer destination near an international airport, in 2019 Port A recorded 204 cruise calls and over 307,000 passengers [20]. A dramatic decline was seen in 2020 due to the pandemic, with Port A recording only 24 cruise calls and less than 20,000 passengers [20]. As an initial approach for responding to the pandemic in 2020, Port A rapidly developed a specific emergency response plan for the management of COVID-19 cases at the port [21]. This initial plan incorporated procedures for case and contact management, without addressing communication procedures with local, national, and competent authorities outside of the country, interoperability of plans with shipping companies, response thresholds, or risk assessment issues. This plan is hereafter referred to as Port A’s “initial COVID-19” PHECP. The suspension of cruise travel and a shortened 2020 cruise season resulted in too few calls at Port A during 2020 to be able to analyze outcomes from the implementation of Port A’s initial COVID-19 PHECP. 

We aimed to review the process of this tourist-based port restarting cruise operations in 2021 supported by COVID-19-focused European and global guidance. We observed the application of this guidance in practice to manage COVID-19 events, and analyzed event responses to recommend revisions to both the port’s PHECP and existing European HEALTHY GATEWAYS guidance for improving preparedness and response to future public health events. 

## 2. Materials and Methods

Port A’s initial COVID-19 PHECP was developed in 2020 as an immediate reaction to the management of COVID-19 cases on board cruise ships. This plan was prepared before the publication of European guidance documents and tools addressing COVID-19 port public health emergency contingency planning [13]. Moreover, the initial plan was developed prior to the issuance of national legislation on public health requirements to be implemented at Greek ports for COVID-19 preparedness and response [22]. As a result, neither the European guidance, nor the national legislation were considered in Port A’s initial PHECP. Therefore, Port A’s initial COVID-19 PHECP was revised before the start of the 2021 cruise season, considering existing gaps and novel COVID-19 components for restarting cruise operations in accordance with European and global guidance. The gaps identified in Port A’s initial COVID-19 PHECP are further described in Table 1.

**Figure 1:**
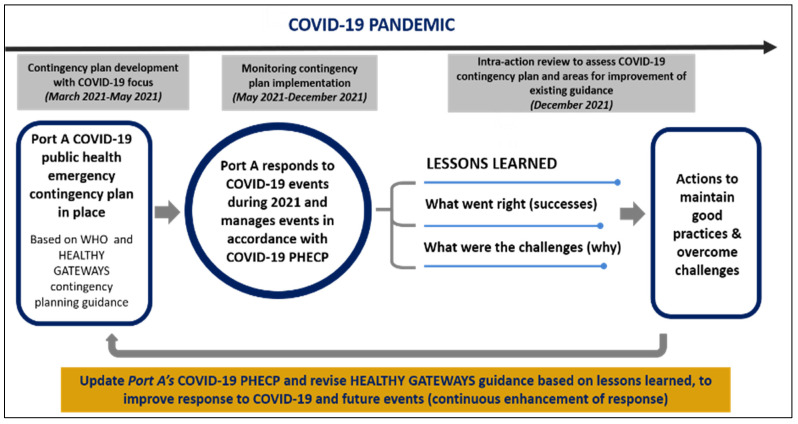
Process of restarting cruise operations and improving response to public health events based on coronavirus disease (COVID-19)-focused contingency planning, and identifying lessons learned from COVID-19 case management at Port A, Greece.

To assess its effectiveness in responding to COVID-19 events, we monitored outcomes from the implementation of Port A’s revised PHECP analyzing epidemiological data during the 2021 cruise season. We further conducted a local IAR reviewing Port A’s COVID-19 response during 2021 to identify good practices, areas for improvement, and lessons learned (Figure 1).

### 2.1. Revision of “Initial COVID-19” Port Public Health Emergency Contingency Plan 

The revision of Port A’s initial COVID-19 PHECP applied the WHO framework for contingency planning at designated points of entry (PoE) [23] and the HEALTHY GATEWAYS tool for assessing port contingency plans in the COVID-19 context [13]. 

Port A established a multidisciplinary core planning team composed of representatives from the port authority, including both senior management and operational personnel, the local health authority, the coast guard, and external maritime public health experts. A structured checklist was developed to identify relevant documentation about the legal framework, operational plans, and processes in which Port A operated, as well as the port profile (available in the Supplementary Material from Table S1: Checklist of relevant documentation). To form the basis of the PHECP, a structured template was produced to (1) define COVID-19 preparedness and response tasks at Port A; (2) identify agencies responsible for each task; and (3) record liaison persons for each responsible agency. Specific tasks were included in the template after considering Port A’s operational plans, service agreements, cruise call itineraries, traveler volumes/origins, and existing COVID-19 guidance [10,12,13,19] (structured template available in the Supplementary Material from Table S2: COVID-19 preparedness and response tasks at Port A). 

With involvement of both regional health and civil protection authorities, the core planning team defined maximum local capacities available for (1) hospitalization of COVID-19 cases, including intensive care unit (ICU) capacities; (2) isolation facilities for COVID-19 cases not requiring hospitalization; (3) quarantine facilities for close contacts of COVID-19 cases; (4) diagnostic laboratory testing facilities for SARS-CoV-2; and (5) transportation of COVID-19 cases and close contacts. Country-level capacities were considered in the plan. This included alternate Greek ports of call with capacities to respond to COVID-19 events, as defined by central level authorities. Templates to monitor capacities were drafted in the plan. During this phase, the planning team included threshold levels at which consideration would be given to stopping cruise calls in a coordinated manner, allowing for repatriation of passengers and crew. Maximum port capacities were decided based on Port A’s 2021 cruise schedule, availability of passenger terminal facilities, and berthing piers for COVID-19 management. The revised plan specified the number of cruise ships for which Port A could function as a home port simultaneously. It further described scenarios for event management if multiple cruise ships calling at Port A experienced COVID-19 events at once. 

During the 2020 cruise season and under the EU joint action HEALTHY GATEWAYS framework, competent authorities from Port A and other Greek ports of call provided epidemiological data to HEALTHY GATEWAYS based on information reported in the Maritime Declarations of Health (MDH) they received. Cruise lines also voluntarily provided epidemiological data from cruise voyages to HEALTHY GATEWAYS through the EU Common Ship Sanitation Database [24]. Based on the ECDC case definitions [25] (which were also applied by HEALTHY GATEWAYS and at national level), epidemiological data reported to HEALTHY GATEWAYS included the number of COVID-19 cases (including if symptomatic), how the case was detected, number of contacts, and follow-up of contacts. Epidemiological data provided to HEALTHY GATEWAYS is currently unpublished, with a manuscript in preparation [26]. Based on epidemiological data collected during the 2020 cruise season through HEALTHY GATEWAYS, the incidence was estimated as 1 COVID-19 case per 1000 travelers (passengers and crew) and approximately 10 contacts per 1 COVID-19 case. Using Port A’s 2021 cruise schedule, we then calculated the number of expected travelers (passengers and crew) calling at the port monthly as a home port. Using the expected incidence, we were able to make hypotheses and estimate the number of expected COVID-19 cases and contacts per month at Port A that might require isolation and quarantine facilities.

Predicted isolation and quarantine facility requirements per month were also calculated for a “worst case scenario” considering 10% of travelers on board cruise ships were infected and required management at their home port. Based on epidemiological data voluntarily reported by ports and cruise lines in 2020 to HEALTHY GATEWAYS through the EU Common Ship Sanitation Database, it was found that day-of-embarkation screening identified, as a maximum, 10 positive COVID-19 cases among 1000 travelers [24], who would not be allowed to embark the cruise. As a worst-case scenario, where pre-embarkation screening failed to identify COVID-19 cases, it was assumed the missed cases would embark, and the first day of the cruise voyage would begin with 10 COVID-19 cases (infectious persons) on board. Assuming a serial interval number of 4–5 days [27,28,29] and a reproduction number of approximately 3–4 [30,31,32], if 10 infectious cases embarked on the first day of the voyage, after an 8 day cruise, approximately 130–210 travelers would be infected on board. It was also assumed that in a worst-case scenario no COVID-19 prevention or control measures were implemented appropriately.

**Figure 2:**
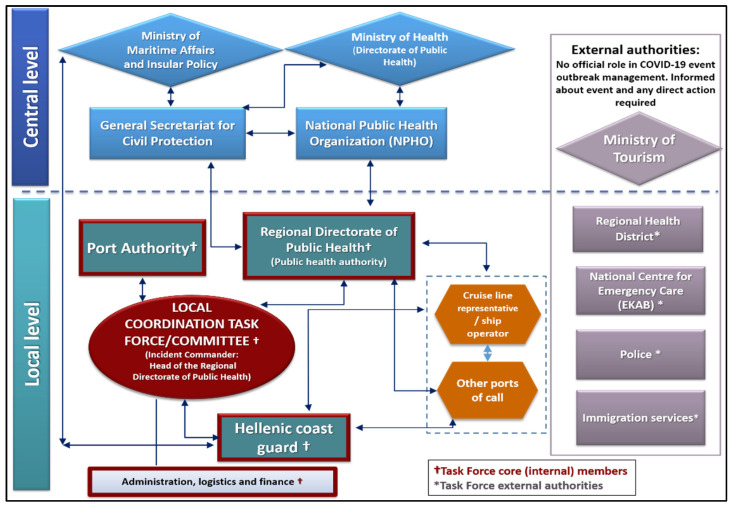
Port A’s organizational structure during activation of the public health emergency contingency plan (PHECP), illustrating the central and local level authorities involved in COVID-19 event management, including the local Task Force members.

The planning team conducted an in-depth review of responsibilities for each COVID-19 preparedness and response task, and finalized the structure of Port A’s local Task Force. This Task Force was responsible for coordinating COVID-19 event management and investigation at port level. It was decided that events presenting small-scale public health risks would trigger activation of Port A’s COVID-19 PHECP and Standard Operating Procedures (SOPs) for case management, while larger-scale events would additionally trigger Task Force activation and requests for central level support. A diagram mapping central and local authorities involved in COVID-19 event management at Port A was developed (Figure 2) to clarify communication between authorities during routine operations and COVID-19 event response. 

### 2.2. Monitoring Implementation of the Revised COVID-19 PHECP during 2021 Cruises

Between May and December 2021, multiple data sources were used to monitor implementation of Port A’s revised COVID-19 PHECP. MDHs submitted to Port A reporting COVID-19 infection on board offered one data source. A positive MDH indicated plan activation and provided epidemiological data about the COVID-19 event. Internal communication records among the Task Force (via WhatsApp mobile application) and formal communications towards external authorities (via email) during COVID-19 event management provided a second source. These communication records allowed for tracking COVID-19 response actions, challenges, and outcomes. As a third data source, local public health capacity levels were regularly available through monitoring records maintained by Port A in cooperation with external authorities. This included monitoring availability of hospital beds for cruise travelers that might require hospitalization, as well as availability of designated facilities (“COVID-19 hotels”) for isolation/quarantine of cruise travelers identified as COVID-19 cases not requiring hospitalization, or contacts of cases when disembarked at Port A. 

### 2.3. Conducting a Local Level Intra-Action Review at Port A during the COVID-19 Pandemic 

A web-based IAR reviewed COVID-19 event management at Port A during 2021 by applying the WHO [16] and ECDC [17,33] methodology for conducting IARs, modified to a cruise port setting. 

The IAR intended to provide key stakeholders an opportunity to discuss response actions, with feedback used to revise the port’s PHECP. Port A’s core multidisciplinary planning team and additional representatives from the regional public health authority, regional public health and environmental hygiene department, local port agency, and local Hellenic coast guard participated. 

To prioritize response areas considered essential to review, a brief telephone pre-interview was conducted with port authority participants. Three COVID-19 events managed by Port A during 2021 were deemed complex and requiring in-depth analysis. The IAR agenda was thus divided into three sessions each analyzing a specific COVID-19 event. Two additional sessions were conducted for good practices during COVID-19 event management and urgent issues for 2022 cruises, respectively. Background information on the three COVID-19 events was requested from the port authority to pre-construct event timelines. Moreover, “discussion templates” were prepared for each IAR session based on WHO and ECDC guidance, with the purpose of facilitating structured analysis of each event (available in the Supplementary Material from Figure S1: Example of COVID-19 event timeline for IAR and Table S3: IAR discussion templates). 

External maritime public health experts participated in the IAR as facilitators and each session followed WHO’s debrief format [34] with facilitated group discussions. Sessions began with the facilitation team presenting an overview of the pre-constructed COVID-19 event timeline. Participants then discussed if the timeline accurately represented the reality of the event, reflecting on key actions and communications. Facilitators led participants through structured analysis, using the discussion templates to identify event challenges and good practices, their impacts, and causal factors. 

## 3. Results

### 3.1. Revision of Port A’s “Initial COVID-19” Plan and Integration of Novel COVID-19 Components 

Assessing Port A’s initial COVID-19 PHECP against contingency planning guidance from HEALTHY GATEWAYS [11,12,13] and WHO [10,23] in the context of COVID-19 identified elements for port preparedness and response that were not addressed in the “initial COVID-19” plan. Furthermore, challenges that emerged during event management in 2020 revealed missing elements from existing HEALTHY GATEWAYS contingency planning guidance for restarting cruise operations. Table 1 describes the gaps in Port A’s initial COVID-19 plan, new elements incorporated into Port A’s revised COVID-19 PHECP, and if these elements were addressed in existing guidance for COVID-19 port public health emergency contingency planning. 

**Table 1:** Gaps in Port A’s “initial COVID-19” PHECP, new elements incorporated into the revised COVID-19 PHECP, their value for preparedness planning, and if elements were addressed in existing guidance.

Gaps Identified in “Initial COVID-19” Plan	Elements Included in Port A’s Revised COVID-19 PHECP	Element Description	Added Value for Preparedness Planning	Element Addressed in Existing European HEALTHY GATEWAYS Guidance
Phased activation of “pre-pandemic” plan	Formal alert phases for activating PHECP and Task Force	Alert phases color-coded by public health risk level. Each phase associated with specific scenarios and response actions	Defines specific conditions for: - Routine preparedness - Activating PHECP and COVID-19 case-management protocols - Activating local Task Force - Requesting support from central level authorities	Addressed in existing guidance
Documented procedures for managing COVID-19 events	Standard Operating Procedures (SOPs)	SOPs for COVID-19 management (cases detected on board or at port) from notification, to management of cases, contacts and health measures implementation	Describes for each involved stakeholder step-by-step procedures, promoting coordinated and timely event response	Addressed in existing guidance
Documented procedures for internal and external information exchange/communication	Communication plan for: (a) routine operations in COVID-19 context; (b) COVID-19 event management	- Internal communication means & routes - Communication means & routes with external stakeholders - Organizational chart defining information flow during event management, from event notification to closing	Supports operational communication in both routine circumstances and COVID-19 event management, to promote coordinated responses from internal and external stakeholders	Addressed in existing guidance
Interoperability of port’s and cruise ships’ COVID-19 response plans	Monitoring framework for alignment of ships’ COVID-19 PHECP with Port A’s PHECP	Table recording for each ship call: - Ports’ role: home, transit or contingency port ^1^ - Receipt of ship’s PHECP (when home/contingency port) or written assurance statement (when transit port) - If plan interoperability ensured	- Pre-defines ports’ role in event management for all calls, for port to take appropriate preparedness measures - Helps determine number of cruise ships for which port can function as home/contingency port	New method to monitor interoperability—will be incorporated in future version of guidance
Identification of responsible authorities for COVID-19 event management	Roles and responsibilities for preparedness and response	- Describes COVID-19 preparedness and management tasks - Records responsible agency for each task and contact persons for each agency	- Cleary defines tasks required for event preparedness and management, including those responsible to conduct tasks - Information readily available during event management	Partially addressed in existing guidance—will be revised in future version to include tasks for monitoring public health capacities
Framework to define and monitor local and country level public health response capacities for COVID-19 event management	Templates defining maximum public health capacities	Templates documenting maximum capacities available for: - Hospitalization (including ICU care) - Isolation facilities - Quarantine facilities - Transportation (ambulance) - Transportation (to isolation/quarantine facilities)	- Informs preparedness planning and helps port determine number of cruise ships for which port can function as home/contingency port	Capacity templates will be incorporated in future version of guidance
	Templates monitoring current public health capacities against pre-defined maximum	Monitoring template includes: - Date of reporting - Maximum capacity available - Current capacity available - % of maximum capacity reached - Agency responsible to monitor capacity	- Regular monitoring (daily/weekly) to provide overall picture of existing response capacities at specific time points to inform risk assessment and decision making - Monitoring and notification when pre-determined threshold of maximum capacity reached, to allow for immediate consultation about possibly stopping cruise calls	Monitoring templates will be incorporated in future version of guidance
Defining local capacities for diagnostic laboratory testing (SARS-CoV-2)	Pre-identification of reliable shore-side laboratories	List of reliable shore-side laboratories performing reverse transcription—polymerase chain reaction (RT-PCR): - Name/location - Contact details of liaison persons - Operating hours	- Defines available local diagnostic testing capacity and helps determine ports’ capacity to act as home/contingency port - Ports can provide list to cruise lines if needed to conduct or confirm diagnostic test results	Partially addressed in existing guidance—future version of guidance to advise that port PHECP should include written agreements with reliable shore-side laboratories
Understanding isolation and quarantine facility requirements for home port calls	Predicted shore-side isolation and quarantine facility requirements during routine and “worst case” scenarios	- Template to estimate expected number of travelers arriving monthly (home port calls) - Template to estimate monthly isolation and quarantine facility requirements - Template to estimate monthly isolation and quarantine facility requirements in “worst case” (10% travelers on board COVID-19 positive)	- Allows port to predict approximate isolation and quarantine facility requirements (as home port) to inform preparedness planning	Templates will be incorporated in future version of guidance
Determining capacities to facilitate repatriations, crew changes etc.	Awareness of local/regional airport/airline operations	- List of airlines operating flights, their destinations and season of operation - List of domestic and foreign aircraft operators with contact details	- Supports planning to define capacities for repatriation and crew changes - Information readily available when needed to support event management	Addressed in existing guidance
Documented agreement of all stakeholders regarding their role in PHECP	Memorandum of Understanding (MoU) among stakeholders	- MoU signed by each authority defined as responsible for a task in the PHECP	- Sets out framework of cooperation between authorities involved in event management; signature confirms agreement with respective responsibilities and mutual understanding	Addressed in existing guidance

^1^ Definition of port categories based on HEALTHY GATEWAYS guidance [13]. Home port: where cruise ship passengers embark to start the cruise and disembark at the end. Should fulfill the criteria of a contingency port; each ship should have at least one contingency port as part of a seven-night itinerary. The home port should always be the contingency port, but additional contingency ports should be defined. Contingency port: interoperability of the ship’s contingency plan and port’s contingency plan has been ensured, and agreed that any potential COVID-19 outbreak on board this cruise ship will be managed at this port, including complete evacuation of the cruise ship if needed and isolation/quarantine of cases/contacts. Transit port: an intermediate stop on itinerary, where passengers will get on/off for excursions. Embarkation at transit ports is allowed provided all relevant measures are applied. A transit port must prepare their port COVID-19-PHECP and be ready to accommodate Category 4 (emergency evacuation) cases when necessary.

The port authority’s senior level management prioritized the immediate allocation of funding to develop the revised COVID-19 PHECP including essential infrastructure, equipment, and human resources, in addition to funding required for execution of the plan during the 2021 cruise season (for example, implementation of preparedness measures at Port A). Furthermore, senior management facilitated engagement of other stakeholders at local, regional, and country level in the planning process, to identify information required for preparing the revised PHECP. This included information about maximum response capacities at local and country level (hospitalization, isolation, and quarantine facilities, transport services, diagnostic laboratory testing, flight operations for repatriations/crew changes), to determine the maximum number of cruise ships for which Port A could make agreements to act as a home port and/or contingency port. Local- and country-level response capacities were expected to fluctuate during the 2021 cruise season as local communities and land-based tourism were also expected to consume capacities. Therefore, the port authority’s senior management agreed to implement a system in the revised COVID-19 PHECP for regularly monitoring capacities and defining “threshold levels” where consideration would be given to stopping cruise calls. With this system, the Task Force could identify existing capacity levels at any point in time and determine if they could support health measures implementation that would be required. As multiple external authorities would provide information to Port A about existing capacity levels, the port’s senior management ensured that the revised COVID-19 PHECP included agreements with these authorities that defined the frequency (weekly) and means (via email, telephone) of capacity updates.

Predicting the number of COVID-19 cases and close contacts Port A expected to receive during 2021 in both routine and worst-case scenarios informed response planning. Since Port A’s role in public health event management was pre-defined in the COVID-19 PHECP for every cruise call in their 2021 schedule, these scenarios helped the core planning team foresee approximate capacity requirements for hospitalization, accommodation in shore-side isolation and quarantine facilities, as well as transportation. Port A could then share predicted capacity needs with relevant local authorities such as health and civil protection, as well as central level authorities for their preparedness planning. Monitoring templates included in the revised COVID-19 PHECP could be regularly updated by the core planning team to reflect changing cruise schedules. 

To facilitate interoperability between Port A’s PHECP with cruise ship response plans, it was agreed with the cruise lines calling Port A that prior to operations, each cruise ship where Port A acted as home port would submit their contingency plan for review. Cruise ships that submitted their contingency plans to a port outside of Greece could provide a written assurance statement (signed by the home competent authority) confirming the ship’s plan was reviewed for interoperability with the home port outside of Greece.

To ensure that capacities for managing a COVID-19 event or outbreak would be available to cruise ships, Port A’s revised PHECP included a list of alternate ports in Greece, where a ship could be asked to divert for case management if Port A determined they did not have the capacities to adequately respond. Alternate ports were based on ports defined by central level authorities as having the ability to act as home or contingency ports considering their capacities. The revised COVID-19 PHECP also included templates for Port A to rapidly communicate with and ensure alternate ports had required capacities to manage an event. 

### 3.2. Implementation of Port A’s Revised COVID-19 Public Health Emergency Contingency Plan 

No COVID-19 outbreak was reported during the study period. Between 11 May and 31 December 2021, Port A recorded 119,930 cruise passengers among 118 calls and 23 cruise ships. From these cruise calls, Port A activated their COVID-19 PHECP and managed a total of 29 cases, originating from 11 cruises on board 7 different cruise ships as detailed in Table 2. Furthermore, no COVID-19 cases were detected at the port facility during this period. Port A was the designated home port for three COVID-19 events; for five events where Port A provided case management, their designated role was as a transit port. A more detailed description of COVID-19 cases recorded by Port A during the study period can be found in Table 3 [20]. It should be noted that of the 29 cases managed by Port A, only 14 were true COVID-19 cases. As indicated in Table 2 and Table 3, 15 asymptomatic individuals were detected as COVID-19 cases on board one cruise voyage, when testing was conducted in a shore-side laboratory at a previous port of call. Upon arrival at Port A for event management, considering the higher-than-expected number of cases, the 15 individuals were re-tested for verification and all were confirmed as false positive. 

In accordance with national legislation (based on HEALTHY GATEWAYS guidance), during the study period of May to December 2021 health screening of cruise passengers was carried out prior to embarkation [12,22]. Passengers underwent pre-boarding screening which included completion of a pre-boarding health declaration questionnaire. Diagnostic test results for entry into the country were checked and passengers also underwent contactless temperature measurement. A measurement above 38° or a positive answer to the pre-boarding declaration led to secondary screening. If a possible case was suspected, passengers and their travelling companions would be tested for COVID-19. Moreover, diagnostic testing of passengers was conducted on the day of embarkation, either via reverse transcription–polymerase chain reaction (RT-PCR) or rapid antigen detection test (RADT). Throughout the study period, health monitoring of passengers on board was proposed to facilitate early detection of symptomatic COVID-19 cases in accordance with HEALTHY GATEWAYS guidance [12]. This included daily contactless temperature measurements; those with measurements of 38° or greater were advised to immediately self-isolate and report symptoms to medical personnel on board. Diagnostic testing (RADT or RT-PCR) was also suggested for all passengers, beginning on the third day of the cruise, and this could be conducted in combination with necessary disembarkation testing for voyages of less than 8 days [12]. 

Case definitions used for surveillance were based on those of ECDC during the study period [35]. It was recommended to initiate case finding following the detection of a possible or confirmed COVID-19 case on board [12]. A symptomatic traveler meeting the clinical criteria of a possible case was isolated and tested for COVID-19 via RADT (if RADT results were negative it was suggested to perform a RT-PCR). If confirmed as a COVID-19 case, the traveler was transported to a medical/isolation facility ashore. Close contacts of a possible case remained on board in quarantine cabins until laboratory results of the possible case were available. If results were positive, close contacts disembarked and quarantined in facilities ashore with active monitoring by public health authorities for 10 days from their last exposure, in addition to daily monitoring for COVID-19 symptoms and avoidance of social contact and travel.

During COVID-19 event management the exchange of information and communication, between Port A’s Task Force, the cruise ships, ports of call, and other authorities, was rapid and complex. It was challenging to understand the timeline of key actions in response to a COVID-19 event using only available internal and external communication records, supplemented with epidemiological data reported in positive MDHs. This highlighted the need for a standard method to immediately record actions taken by authorities involved in COVID-19 event response, to provide a basis for facilitating the identification of lessons learned at a future point. Thus, an additional element incorporated into Port A’s PHECP is a standardized event reporting template, to be completed in real-time and provide a record of response actions during each stage of event management.

### 3.3. Results from Port A’s Intra-Action Review to Analyze COVID-19 Event Response 

The in-depth qualitative analysis of Port A’s COVID-19 response through the IAR highlighted gaps in the port’s revised COVID-19 PHECP and existing European guidance. Diverse opinions during analysis of response actions were provided through participation of the core multidisciplinary planning team and other authorities. Participants presented their experiences from the perspectives of public health, port operations, port security, and ship agencies. Challenges and good practices identified generated action points for improving port preparedness and response at European, national and port level. The facilitation team prepared a final report of action points and shared with participants for validation after the IAR concluded. Following validation, IAR findings and action points were shared with the relevant national authorities for tourism and maritime affairs, as well as European level agencies. An evaluation survey based on WHO guidance [16] was developed and shared with review participants to gain insight on how the conduct of future IARs could be improved.

Table 4 describes for each IAR session the identified challenges and good practices, as well as recommended revisions to Port A’s COVID-19 PHECP and existing EU HEALTHY GATEWAYS guidance for contingency planning, preparedness and response.

As described in Table 4, a challenge identified during multiple events was requests for Port A to provide COVID-19 event management in non-emergency situations, even though Port A was designated as a transit port in the ship’s itinerary. Given the frequency of this situation, it was agreed that Port A must conduct a risk assessment, which also emerged as a good practice they had implemented throughout 2021. This risk assessment considered (1) existing port capacities (availability of berthing piers, port isolation facilities) and duration of availability; current public health capacities (for hospitalization, isolation, quarantine, diagnostic laboratory testing) and duration of availability; (2) risk of the COVID-19 event to the port and local community, considering number of cases and close contacts, the health and vaccination status of travelers; and (3) feasibility of event management (considering distance of ship from its home port, local flight operations available for repatriation etc.). To ensure this practice continues to be implemented by transit ports, it was recommended that the practice be formally documented in both Port A’s PHECP and considered in future contingency planning guidance from HEALTHY GATEWAYS. It was also recommended to formally document in Port A’s PHECP that risk assessments will be carried out in situations where a COVID-19 event exceeds what is expected or probable, considering the epidemiological situation. Analysis of specific COVID-19 events during the IAR indicated that conducting a risk assessment as soon as the event was detected could have prevented implementation of health measures such as disembarkation, isolation/quarantine, and registration in the national COVID-19 database of 15 cases that were later verified as false positive.

During management of COVID-19 events onboard ships calling at both European and non-European countries in a single itinerary, limited communication was a challenge identified. As outlined in Table 4, limited communication existed between Port A and the next port of call in European and particularly non-European countries. Lack of information exchange between port level authorities led to challenges, where health measures recommended by Port A were not implemented by the cruise ship once leaving European waters. Direct communication between Port A and non-European port authorities via informal channels was recommended at this stage. However, the IAR identified an urgent need to facilitate communication between cruise ports in European and non-European countries, since COVID-19 public health events occur on board cruise ships calling both in a single itinerary.

The IAR also identified challenges that arose due to the possibility of differing response protocols implemented by ports of European and non-European countries for COVID-19 event management. For example, in accordance with national protocols and HEALTHY GATEWAYS guidance, with detection of one COVID-19 case on board, Port A would disembark cases and close contacts for shore-side hospitalization/isolation/quarantine, with passengers disembarked for repatriation as per competent authority instructions. The IAR identified an assumption that passenger disembarkation for repatriation would not have been allowed by the non-European country if a COVID-19 case was identified on board, with the possibility of shore-side quarantine of all passengers. As a result of anticipating different or potentially more stringent response measures, the cruise ship did not continue to the port of disembarkation in a non-European country, but returned to Port A as the previous port of call for repatriation of passengers (event described in Table 4). This lack of coordinated response standards revealed an urgent need to establish shared protocols for COVID-19 public health response at cruise ports between European and non-European countries. A recommendation emerging from the IAR was that central level competent authorities could communicate directly with non-European countries included in cruise itineraries, for establishing coordinated responses to public health events onboard cruise ships among local ports. 

## 4. Discussion

Country-led IARs have been conducted globally to assess several COVID-19 response domains (pillars) simultaneously, including a pillar for points of entry (PoE) [36,37,38]. IARs have also been implemented to focus specifically on countries’ COVID-19 response at PoE [39,40], with some IARs for ports and airports carried out under the European HEALTHY GATEWAYS framework [41,42]. However, to the best of our knowledge this is the first published study applying IAR methodology to a single point of entry at local level, to assess effectiveness of port-specific contingency planning and integrate lessons learned for rapid revision of the port’s PHECP and existing European guidance. 

Findings from an IAR assessing Vietnam’s PoE surveillance system early in the COVID-19 pandemic identified preparedness gaps stemming from inadequate implementation of plans already in place, since there were no conditions to enforce plans [40]. Implementation of the revised COVID-19 PHECP was not identified as a challenge at Port A. The port authority’s senior management was engaged throughout the process of revising, implementing, and assessing Port A’s COVID-19 PHECP. Their prioritization of preparedness activities proved to be a useful strategy for contingency planning. Decisions could be taken swiftly during plan development/implementation, and recommendations emerging from the IAR could be implemented immediately to improve Port A’s response. This is in line with a good practice repeatedly observed in IARs assessing various COVID-19 response areas, where involvement of senior leadership and informed personnel supported rapid decision making and actions [43]. Defining roles and communication structures of Port A’s multi-disciplinary Task Force also allowed for efficient risk assessment and decision-making by the different agencies involved during COVID-19 events. The value of a multi-agency team for rapid assessments and decision-making was similarly reported in a study analyzing the Port of Hamburg’s organizational processes in the context of mass casualty incidents from infectious diseases [44].

Multisectoral cooperation is required to manage public health events at PoE, with involvement of multiple authorities, agencies and service providers across sectors [23,45]. The WHO Joint External Evaluation tool also emphasizes development of a “multisectoral” public health emergency contingency plan in the context of public health response at PoE [46]. A recurring challenge identified in the literature both before and during the COVID-19 pandemic was lack of clarity regarding roles and responsibilities for managing public health events at PoE and on conveyances, highlighting the need to clearly document the roles and responsibilities of all stakeholders involved [1,40,45,47]. A similar challenge was observed when revising Port A’s initial COVID-19 PHECP, as limited experience managing COVID-19 events during 2020 made it difficult to identify necessary COVID-19-preparedness and response tasks. After establishing a framework to categorize tasks (available in the Supplementary Material from Table S2: COVID-19 preparedness and response tasks at Port A), an essential step by the core planning team was discussing each task individually until a consensus was reached to identify the responsible authorities. This helped to quickly identify and clarify uncertainties about roles at central, regional, and local levels.

Communication challenges observed during the IAR reflect results of a study under the EU SHIPSAN project examining passenger ship hygiene inspection practices, where a lack of port-to-port communication was identified [48]. In a review of reports from COVID-19 IARs conducted in WHO African region countries until March 2021, the importance of appropriate information exchange between neighboring countries in the PoE context emerged [38]. COVID-19 revealed the importance of timely communication between ports in a cruise ship’s itinerary, including European and non-European countries. Existing guidance encourages information exchange at port level within and between countries, to inform about health measures implemented and recommended in response to COVID-19 events on board [12]. As countries may have differing communication regulations and practices, formal and existing communication channels are essential. Use of existing platforms facilitating port-to-port communication could be explored [49], particularly to support information exchange between ports in European and non-European countries. 

Lack of standardized responses to COVID-19 events on board ships, observed early in the pandemic [8,50] and discussed during the IAR at Port A, highlighted the need for common public health response protocols as ships continuously sail between European and non-European countries. A possible solution to be explored is the development of a regional program of collaboration between ports of specific European and non-European countries for implementation of shared protocols, as well as education and training on these protocols. 

Regular testing of port PHECPs through exercises is recommended to assess plan functionality, contribute to continuous improvement, and provide an opportunity for training [23,44,45,46]. In the context of COVID-19, it may be challenging to organize exercises, given limited human resources and time constraints. The IAR methodology provides a feasible alternative to rapidly assess port PHECPs and document lessons learned, while experiences from COVID-19 are recent and not as difficult to recall. This methodology introduced by WHO [16] and ECDC [17,33] is highly flexible for application to unique settings such as ports. We recommend countries implement short IARs at the level of a single point of entry, to facilitate in-depth reviews of response areas modified to the PoE setting, such as port-level coordination/monitoring, case management, risk communication, or other areas based on local needs. These smaller-scale IARs could promote immediately implementable actions, and common challenges to provide the basis for repeated IARs as recommended by WHO [16]. In the future, focused IARs analyzing a specific COVID-19 event involving several ports could be conducted with participation of European and non-European countries, to review COVID-19 responses transnationally. Applying an “embedded” IAR approach [51] may also be considered for the point-of-entry setting, where maritime public health experts could observe operations at the PoE, to gain a better understanding of challenges and good practices implemented during response to COVID-19 events. 

As the epidemiological situation evolves and restrictive measures implemented in response to COVID-19 on cruise ships are relaxed, cases and outbreaks may still occur on board. It will be important to have in place COVID-19 port PHECPs for case management or response to future outbreaks. However, to prevent COVID-19 and support preparedness for future public health threats, it may also be valuable to integrate measures for COVID-19 prevention and control into routine cruise ship and port operations. Lessons identified through COVID-19-focused IARs can be used to review and enhance existing prevention and control measures, to be incorporated into cruise ships’ and ports’ preparedness plans. IARs could become a routine measure when a COVID-19 event occurs onboard a cruise ship or at a port during and after the pandemic. This could be a means to quickly and regularly assess response actions, prevention, and control measures. Since IARs could be a useful tool during future prolonged public health emergencies, gaining experience in conducting IARs at national, regional, and port level is considered valuable. 

Following the IAR at Port A, a European level meeting was conducted under the HEALTHY GATEWAYS framework, applying the IAR methodology to analyze a COVID-19 outbreak on board a cruise ship [52]. This meeting allowed central and local stakeholders, from different European countries, as well as cruise line representatives, to share their COVID-19 response experiences, discuss lessons, and exchange good practices. WHO also explicitly supports the sharing of IAR results between countries to facilitate an exchange of good practices [16]. Looking to the future, specific workshops among ports at European or global level could be organized to share good practices and lessons learned from COVID-19-focused IARs. 

While most gaps in Port A’s PHECP were elements detected through existing HEALTHY GATEWAYS [11,12,13], ECDC-EMSA [19], and WHO [10,23] guidance, the revision process identified missing elements which we propose should be incorporated into upcoming versions of HEALTHY GATEWAYS guidance and future public health emergency contingency planning guidance for ports. Future guidance must focus on addressing how to monitor port capacities and interoperability of the port PHECP with cruise ship plans, as well as how to define and monitor both local and country capacities for public health event management. It should also emphasize the importance of ensuring port PHECPs have written agreements with authorities for monitoring capacities, and written agreements with reliable shore-side diagnostic laboratories. To enhance preparedness capacities, future guidance could also provide tools to support ports with determining capacity needs (for example, isolation and quarantine facilities) based on the expected volume of travelers calling at the port. 

A limitation of the study is that recommendations described are based on the experience of only one port in a tourist context with a unique risk profile. However, we have seen from reported COVID-19 outbreaks on passenger ships that the pandemic presented common challenges to port authorities for event management. Particularly at the start of the pandemic, these included challenges with identification and arrangement of isolation facilities and transport means [53], limited diagnostic laboratory testing capacities [47], and lack of feasibility for shore-side quarantine and transport of mass evacuations [47,54]. Complex lines of command between the different authorities involved in COVID-19 event response were seen [54]. Furthermore, a report commissioned by the Japanese Ministry of Foreign Affairs to explore prevention and response to infectious disease outbreaks on cruise ships identified, as a major challenge, the lack of clarity regarding responsibility for event management [47]. Port A’s experiences identified elements that could be integrated in future European and global guidance for ports’ public health emergency preparedness. Lessons learned can be applied to other ports, to promote common standards for public health event management while preventing adverse and variable responses to future COVID-19 events or other future health emergencies. 

## 5. Conclusions

The COVID-19 pandemic challenged effective response to events on board cruise ships and at ports, with inadequate public health response capacities and lack of clarity on roles and responsibilities for event management observed. This reflects the need for pre-determined, harmonized port PHECPs where lessons learned from unprecedented COVID-19 experiences can be immediately integrated to strengthen plans. Existing European and global port contingency planning guidance, as well as novel components identified through Port A’s experience revising their own plan, can address these challenges and improve preparedness plans. Using WHO and ECDC methodology to conduct IARs at local port level is a feasible method to assess the effectiveness of PHECPs, while contributing to their continuous improvement for response to COVID-19 and future unknown emerging threats. 

## Figures and Tables

**Table 2:** COVID-19 events ^1^ managed by Port A in accordance with the revised PHECP between May to December 2021.

Date of Call (Month/Year)	Cruise Ship	Total Number of Passengers	Passengers at Arrival	Number of COVID-19 Cases ^2^	Number of Close Contacts ^3^	Port A’s Designated Role in Itinerary
05/2021	Cruise ship A	1971	949	1	5	Home port
05/2021	Cruise ship A	2313	1316	2	2	Home port
08/2021	Cruise ship B	155	88	1	1	Transit port
09/2021	Cruise ship C	408	403	1	1	Transit port
09/2021	Cruise ship D	1335	1335	1	0	Transit port
10/2021	Cruise ship E	457	455	2	2	Transit port
10/2021	Cruise ship A	2284	1258	3	0	Home port
10/2021	Cruise ship D	1278	1278	1	1	Transit port
11/2021	Cruise ship F	1135	1134	1	1	Transit port
11/2021	Cruise ship G	117	115	1	0	Transit port

^1^ An additional event not included in Table 2 was managed by Port A, where 15 individuals (all asymptomatic) were detected as COVID-19 cases via reverse transcription—polymerase chain reaction (RT-PCR) conducted at a shore-side laboratory in a previous port of call (testing conducted as a requirement for entry into another country later in the cruise itinerary). During event management at Port A and upon re-testing for verification, all 15 individuals were confirmed as false positive. ^2^ Detection of COVID-19 cases varied; in some instances, COVID-19 cases were detected via RT-PCR, and in other instances cases were detected via rapid antigen detection test (RADT) and confirmed via RT-PCR. ^3^ The definition of close contact is based on the European Union (EU) joint action HEALTHY GATEWAYS definition applied in 2021 [12].

**Table 3:** COVID-19 cases and close contacts recorded by Port A during the study period ^1^ (May to December 2021).

Event Number	Cruise Ship Name	Illness	Number of Cases	Passenger or Crew	Symptom Status	Disembarkation Location	Number of Close Contacts ^2^	Number of Crew	Number of Passengers
1	Cruise ship A	COVID-19	1	Passenger	Symptomatic	Port A	5	745	958
2	Cruise ship A	COVID-19	1	Passenger	Symptomatic	Port A	1	750	1316
	Cruise ship A	COVID-19	1	Passenger	Symptomatic	Port A	1	750	1316
3	Cruise ship B	COVID-19	1	Passenger	Symptomatic	Port A	1	Not known	Not known
4	Cruise ship D	COVID-19	1	Crew member	Symptomatic	No disembarkation	Not known	Not known	Not known
5	Cruise ship D	COVID-19	1	Crew member	Asymptomatic	No disembarkation	1	Not known	Not known
6	Cruise ship A	COVID-19	1	Passenger	Symptomatic	No disembarkation	0	789	1258
	Cruise ship A	COVID-19	1	Passenger	Symptomatic	No disembarkation	0	789	1258
	Cruise ship A	COVID-19	1	Passenger	Symptomatic	No disembarkation	0	789	1258
7	Cruise ship F	COVID-19	1	Passenger	Symptomatic	Port A	1	Not known	Not known
8	Cruise ship G	COVID-19	1	Passenger	Asymptomatic	No disembarkation	Not known	444	115
9	Cruise ship C	COVID-19	1	Not known	Not known	Not known	1	Not known	Not known
10	Cruise ship E	COVID-19	2	Not known	Not known	Not known	2	Not known	Not known

^1^ An additional event not included in Table 3 was managed by Port A, where 15 individuals (all asymptomatic) were detected as COVID-19 cases via RT-PCR conducted at a shore-side laboratory in a previous port of call (testing conducted as a requirement for entry into another country later in the cruise itinerary). During event management at Port A and upon re-testing for verification, all 15 individuals were confirmed as false positive. ^2^ The definition of close contact is based on the EU joint action HEALTHY GATEWAYS definition applied in 2021 [12].

**Table 4:** Challenges, good practices, proposed revisions to Port A’s COVID-19 PHECP and revisions to existing EU HEALTHY GATEWAYS guidance based on intra-action review (IAR) findings.

Challenges, Successes, and Recommendations to Improve COVID-19 Response	Description of Findings
Challenges:	Port A designated as transit port but functioned as home port for public health event management, due to inability of other contingency ports to deal with events ^1,2,3^
	Other ports of call in cruise itinerary within the country lacked preparedness plans and capacities for COVID-19 event management ^3^
	Considered less feasible for cruise ship with event on board to return to home port for event management ^1,2^
	Limited/no communication with next port of call in non-European country ^1^
	Lack of formal communication channels between cruise ports of European and non-European countries ^1^
	Cruise operator possibly unfamiliar with response plans in non-European country, or anticipated non-European port may implement different/more rigid COVID-19 response measures ^1^
	Lack of coordinated and common standards for COVID-19 event management between cruise ports of European and non-European countries ^1^
	Health measures (disembarkation, shore-side isolation/quarantine) at Port A implemented before verification of diagnostic test results ^2^
	Limited alternatives and time to verify diagnostic test results in shore-side laboratory before cruise ship arrival at Port A and implementation of health measures ^2^
	Verification of diagnostic test results revealed all COVID-19 cases false positive, but travelers characterized as COVID-19 cases in national case registry database ^2^
	Difficulties in declassifying false positive cases in the national case registry database, in order for travelers to continue their voyage ^2^
	Diagnostic testing conducted on board cruise ship cannot be recorded in national case registry, thus false positives cannot be declassified using test results conducted on board ^2^
	Health measures recommended by Port A authorities were not implemented by the cruise ship after leaving European waters for next port of call in non-European country ^2^
	On the tourist-based island, designated “COVID-19” hotels for isolation/quarantine facilities do not operate in winter season, meanwhile Port A continues to receive cruise calls during winter ^4^
Good practices:	Port A communicated directly with cruise ship and requested verification of event before decision-making ^1^
	Port A conducted risk assessment before managing COVID-19 event as a transit port, considering: current port and local public health capacity levels; risk that event presents to port; feasibility of ship returning to home port ^1,2,3^
	Port A’s PHECP included list of reliable shore-side laboratories with capacity for RT-PCR, to verify unexpected diagnostic test results ^2^
	Joint Ministerial Decision passed at national level, defining process to declassify COVID-19 cases in the national case registry database, defining situations where declassification is permitted ^2^
	In situations where state designated COVID-19 hotels are available for ashore isolation/quarantine, local public health authority insists cases/close contacts are accommodated there, rather than other options (e.g., AirBnB), since designated facilities have capacity to monitor health measures implementation ^4^
Additional revisions proposed for Port A’s COVID-19 PHECP:	Port A will conduct risk assessment in situations when designated as transit port and requested to respond as home/contingency port for non-emergency situations. If results of risk assessment determine that required capacities exist for the duration needed and it is feasible, Port A can activate their PHECP for event management, while informing national authorities and the home port ^1,3^
	Port A communication plan to consider that competent port authority will communicate directly with next port of call in non-European country, informing about COVID-19 event and health measures recommended (via email or telephone) until formal communication channels for information exchange established ^1^
	Describe process for risk assessment prior to health measures implementation when number of COVID-19 cases exceeds expected/probable. Requires verification of diagnostic test results, either retesting via RT-PCR on board or at reliable shore-side laboratory ^2^ Define situations where reclassification of COVID-19 cases may be required, and procedures for Port A to initiate reclassification ^2^
Proposed revisions to EU HEALTHY GATEWAYS guidance:	Describe specific considerations for managing non-emergency COVID-19 events as a transit port and define risk assessment procedures considering: current port capacities; current public health capacities; risk COVID-19 event presents to travelers on board, at port facility and to local port community; other relevant factors ^1,3^
	Contingency planning guidance to include considerations for verification of diagnostic test results (defining risk assessment process) ^2^
	Contingency planning guidance to include cooperation/written agreements with “reliable” shore-side laboratories (if possible accredited and/or certified) ^2^
	Contingency planning guidance to describe scenarios for re-classification of incorrect diagnostic test results in national case registries ^2^
	In certain situations if it is confirmed that no state-designated shore-side isolation/quarantine facilities are available and cruise operators have not arranged other facilities, consider that COVID-19 case may have to remain on board until next port of call, emphasizing preference to disembark case for public health reasons ^4^

Description of COVID-19 events and issues analyzed during the IAR: ^1^ Session 1 event: While en route to next port of call (disembarkation port) in non-European country, request from cruise ship with COVID-19 case on board to return to Port A (previous port of call) for COVID-19 management. ^2^ Session 2 event: Vaccinated passengers underwent RT-PCR testing at shore-side laboratory in previous port of call, with higher-than-expected number of COVID-19 cases detected (all asymptomatic) and characterized in national case registry. Upon arrival at Port A, the port PHECP was activated and health measures were implemented. Verification of test results locally at shore-side laboratory identified all COVID-19 cases as false positives. ^3^ Session 3 event: COVID-19 case identified on board and isolated. Case not disembarked at next ports of call, only disembarked once reaching Port A. ^4^ Session 4—urgent issues: IAR participants identified limitations to isolation and quarantine capacities in tourist-based setting as a considerable challenge for the 2022 cruise season.

## Supplementary Materials

The following supporting information can be downloaded at: https://www.mdpi.com/article/10.3390/ijerph192013262/s1, Table S1: Checklist of relevant documentation; Table S2: COVID-19 preparedness and response tasks at Port A; Table S3: IAR structured discussion templates; Figure S1: Example of COVID-19 event timeline for IAR.
