# Supplementary material for: Re-Starting the Cruise Sector during the COVID-19 Pandemic in Greece: Assessing Effectiveness of Port Contingency Planning
Source: Int J Environ Res Public Health. 2022 Oct 14;19(20):13262. doi: 10.3390/ijerph192013262 (PMC9603745; doi:10.3390/ijerph192013262)
Supplement: Supplementary file 1 [file ijerph-19-13262-s001.zip › TableS1_PHECP_documentation.pdf]

Relevant documentation for COVID-19 public health emergency contingency plan (PHECP) development

<u>Introductory note</u>:

The structured checklist below was prepared to support *Port A's* multidisciplinary core planning team to identify, collect and review relevant documentation prior to revision of the COVID-19 specific port PHECP.

It indicates a suggested** list of documentation to:

- *Inform the planning process*
- *Provide context to the current legal frameworks, operational plans and processes in which Port A operates*
- *Facilitate interoperability of Port A's COVID-19 specific plan with existing health and emergency plans*
- *Further understanding of the port profile of Port A*

Suggested documentation for review addresses different:

1. Levels (local, regional, national, international)
2. Formats (legislation, regulations, policies, guidance documents operational plans, external service agreements)
3. Topics (COVID-19 and other public health risks, emergency/crisis response, maritime/port operations)

**This list is non-exhaustive and can be modified as required.

Relevant documentation	Competent authority	Document title (available from)	Collected	Reviewed	Relevant remarks
International health legislation		e.g. International Health Regulations (IHR) 2005	☐Yes ☐ No	☐Yes ☐ No	
European health legislation		e.g. Decision No 1082/2013/EU on serious cross-border threats to health<br>e.g. Council Directive 2008/114/EC of 8 December 2008 on the identification and designation of European critical infrastructures and the assessment of the need to improve their protection	☐Yes ☐ No	☐ Yes ☐ No	
National legislation on implementing International Health Regulations 2005			☐Yes ☐ No	☐ Yes ☐ No	
National legislation on implementing Decision No 1082/2013/EU			☐Yes ☐ No	☐ Yes ☐ No	
National health legislation/policies			☐Yes ☐ No	☐Yes ☐ No	
National communicable disease legislation/policies			☐Yes ☐ No	☐Yes ☐ No	
National legislation related to COVID-19			☐Yes ☐ No	☐Yes ☐ No	
National maritime legislation/policies			☐Yes ☐ No	☐Yes ☐ No	
National crisis legislation/policies (Civil protection/defense)			☐Yes ☐ No	☐Yes ☐ No	
National emergency management legislation/policies			☐Yes ☐ No	☐Yes ☐ No	
National/regional public health emergency response plans			☐Yes ☐ No	☐Yes ☐ No	
Regulatory agency documents (from customs, biosecurity, police, military)			☐Yes ☐ No	☐Yes ☐ No	
Industry regulations and plans			☐Yes ☐ No	☐Yes ☐ No	

Local maritime regulations and plans			☐Yes<br>☐ No	☐Yes<br>☐ No	
Local public health emergency response plans			☐Yes<br>☐ No	☐Yes<br>☐ No	
Policies and contingency plans from nearby airports (if applicable)			☐Yes<br>☐ No	☐Yes<br>☐ No	
Policies and contingency plans from nearby ground crossing stations<br>(if applicable)			☐Yes<br>☐ No	☐Yes<br>☐ No	
Local legislation related to COVID-19			☐Yes<br>☐ No	☐Yes<br>☐ No	
Local civil protection plan			☐Yes<br>☐ No	☐Yes<br>☐ No	
Operator security plans (framework of Directive 2008/114/EC)			☐Yes<br>☐ No	☐Yes<br>☐ No	
General port emergency plan			☐Yes<br>☐ No	☐Yes<br>☐ No	
Port policy, operational plan, safety plan and emergency plan			☐Yes<br>☐ No	☐Yes<br>☐ No	
Description of port facilities<br>a) port site plan<br>b) safety equipment register<br>c) map of locations<br>d) diagram of port facilities			☐Yes<br>☐ No	☐Yes<br>☐ No	
Service provider operational capability documents and contracts			☐Yes<br>☐ No	☐Yes<br>☐ No	
Port vector management plan			☐Yes<br>☐ No	☐Yes<br>☐ No	
Previous public health or emergency plans for port			☐Yes<br>☐ No	☐Yes<br>☐ No	
Existing "after action" or "post incident" reports/reviews from past port public health responses			☐Yes<br>☐ No	☐Yes<br>☐ No	
Results from WHO Core Capacity Assessment Tool (if applicable)			☐Yes<br>☐ No	☐Yes<br>☐ No	

Statistical data related to the port:<br>a) Number of ship visits and types of ships<br>b) Volume passengers/crew and origin and others (e.g. technicians, harbor pilots, truck drivers etc.)<br>c) Types of cargo handled<br>d) Cruise ship itineraries and previous and next ports of call for ships calling			☐Yes<br>☐ No	☐Yes<br>☐ No	
Guidance on public health, communication disease, international travel, ship and ports prepared by WHO and IMO			☐Yes<br>☐ No	☐Yes<br>☐ No	
Guidance documents related to COVID-19, international travel, ships and ports prepared by global and European agencies			☐Yes<br>☐ No	☐Yes<br>☐ No	
Public health emergency contingency plans (PHECP) of cruise ships calling at port			☐Yes<br>☐ No	☐Yes<br>☐ No	
Cruise ship and port agreements for COVID-19 event management			☐Yes<br>☐ No	☐Yes<br>☐ No	
Any other relevant local rules/regulations			☐Yes<br>☐ No	☐Yes<br>☐ No	
