# Supplementary material for: Re-Starting the Cruise Sector during the COVID-19 Pandemic in Greece: Assessing Effectiveness of Port Contingency Planning
Source: Int J Environ Res Public Health. 2022 Oct 14;19(20):13262. doi: 10.3390/ijerph192013262 (PMC9603745; doi:10.3390/ijerph192013262)
Supplement: Supplementary file 1 [file ijerph-19-13262-s001.zip › TableS2_Task_Definition.pdf]

Roles and responsibilities for COVID-19 preparedness and response

Introductory note:

The structured template below outlines specific responsibilities and tasks required for preparedness and response to COVID-19 events. It was developed to support *Port A's* core planning team to identify and record:

1) *General and specific tasks for COVID-19 preparedness and management at Port A*
2) *Agencies at local/regional/national level responsible for each task*
3) *Contact details of liaison persons for each responsible agency (including alternate liaison person)*

General tasks for the Preparedness phase (A) and Response phase (B) are sub-divided into specific tasks.

For each specific task it is recommended to record the following:

- *Brief task description*
- *Responsible agency (e.g. committee, competent authority, co-competent authority/ service provider)*
- *Liaison person contact details (main contact and alternate contact)*

**Please note this is a non-exhaustive list, and additional tasks can be added or modified.

Preparedness phase (A) tasks are categorized by:

- *1A: Tasks related to defining public health capacities*
- *2A: Tasks related to monitoring public health capacities*

Response phase (B) tasks are categorized by:

- *1B: Tasks for management of a COVID-19 event by Port A*
- *2B: Tasks related to communication during COVID-19 event response*

A. PREPAREDNESS PHASE

1A. Tasks for defining public health capacities

GENERAL TASK A1: Isolation capacities			
**Task no.**	**Specific task description**	**Responsible agency**<br>**(committee, competent authority, co-competent authority/ service provider)**	**Liaison persons**<br>**1. Main contact**<br>**2. Alternate contact**
**A1.1.**	**Arrange pre-designated isolation facilities ashore (e.g. COVID-19 hotels) for cases not requiring hospitalization**	•	1. ....<br>2. ....
		•	1. ....<br>2. ....
**A1.2.**	**Define existing/ maximum capacity available to individually isolate (with their own designated bathroom) confirmed COVID-19 cases**	•	1. ....<br>2. ....
**A1.3.**	**Define maximum threshold of isolation capacities for stopping cruise ship calls**	•	1. ....<br>2. ....

GENERAL TASK A2: Quarantine capacities			
**Task no.**	**Specific task description**	**Responsible agency**<br>**(committee, competent authority, co-competent authority/ service provider)**	**Liaison persons**<br>**1. Main contact**<br>**2. Alternate contact**
**A2.1.**	**Arrange pre-designated quarantine facilities ashore (e.g. COVID-19 hotels) for quarantine of close contacts**	•	1. ....<br>2. ....
		•	1. ....<br>2. ....
**A2.2.**	**Define existing/ maximum capacity available to quarantine close contacts**	•	1. ....<br>2. ....
**A2.3.**	**Define maximum threshold of quarantine capacities for stopping cruise ship calls**	•	1. ....<br>2. ....

**GENERAL TASK A3: Hospitalization capacities**			
**Task no.**	**Specific task description**	**Responsible agency** (committee, competent authority, co-competent authority/ service provider)	**Liaison persons** 1. Main contact 2. Alternate contact
**A3.1.**	**Define/arrange hospitals in local area with capacity to provide care for COVID-19 cases (including ICU care)**	• ……………………….	1. …….. 2. ……..
**A3.2.**	**Define existing and maximum capacity for hospitalization of COVID-19 cases in local area (including ICU care)**	• ……………………….	1. …….. 2. ……..
**A3.3.**	**Define maximum threshold of hospitalization capacities for stopping cruise ship calls**	• ……………………….	1. …….. 2. ……..
**GENERAL TASK A4: Alternate port capacities**			
**Task no.**	**Specific task description**	**Responsible agency** (committee, competent authority, co-competent authority/ service provider)	**Liaison persons** 1. Main contact 2. Alternate contact
**A4.1.**	**Define alternate ports with capacities to manage outbreaks and situations where alternate ports asked to manage public health events**	• ……………………….	1. …….. 2. ……..
**GENERAL TASK A5: Transport capacities**			
**Task no.**	**Specific task description**	**Responsible agency** (committee, competent authority, co-competent authority/ service provider)	**Liaison persons** 1. Main contact 2. Alternate contact
**A5.1.**	**Pre-define arrangements for transferring cases/contacts to isolation/ quarantine facilities**	• ……………………….	1. …….. 2. ……..
**A5.2.**	**Pre-define arrangements for transport means for transferring cases to hospital/medical facility**	• ……………………….	1. …….. 2. ……..

GENERAL TASK A6: Diagnostic laboratory capacities			
**Task no.**	**Specific task description**	**Responsible agency**<br>**(committee, competent authority, co-competent authority/ service provider)**	**Liaison persons**<br>**1. Main contact**<br>**2. Alternate contact**
**A6.1.**	**Identify diagnostic laboratory facilities/capacities for SARS-CoV-2**	• ………………………..	1. ……..<br>2. ……..

GENERAL TASK A7: Preparations for coordination of public health event management and ensuring interoperability with cruise ship contingency plans			
**Task no.**	**Specific task description**	**Responsible agency**<br>**(committee, competent authority, co-competent authority/ service provider)**	**Liaison persons**<br>**1. Main contact**<br>**2. Alternate contact**
**A7.1.**	**Coordinate public health event management and investigation at port level**	• ………………………..	1. ……..<br>2. ……..
		• ………………………..	1. ……..<br>2. ……..
		• ………………………..	1. ……..<br>2. ……..
**A7.2.**	**Define situations where help will be asked from central level authorities**	• ………………………..	1. ……..<br>2. ……..
**A7.3.**	**Review ship's contingency plans/written assurance statements for interoperability with port's PHECP**	• ………………………..	1. ……..<br>2. ……..

A. PREPAREDNESS PHASE

2A. Tasks for monitoring public health capacities

GENERAL TASK A8: Regularly monitoring public health capacities			
**Task no.**	**Specific task description**	**Responsible agency** (committee, competent authority, co-competent authority/ service provider)	**Liaison persons** 1. Main contact 2. Alternate contact
**A8.1.**	**Regular updates about number of available isolation rooms in local area**	• ………………………	1. …….. 2. ……..
**A8.2.**	**Regular updates about number of available rooms at quarantine rooms in local area**	• ………………………	1. …….. 2. ……..
**A8.3.**	**Regular updates about number of available beds to care for COVID-19 cases (including ICU beds) at each facility**	• ………………………	1. …….. 2. ……..
**A8.4.**	**Regular updates on existing capacities available to transport asymptomatic/mild COVID-19 cases or close contacts to isolation or quarantine**	• ………………………	1. …….. 2. ……..
**GENERAL TASK A9: Training activities**			
**Task no.**	**Specific task description**	**Responsible agency** (committee, competent authority, co-competent authority/ service provider)	**Liaison persons** 1. Main contact 2. Alternate contact
**A9.1.**	**Training of staff and verification of training**	• ………………………	1. …….. 2. ……..
**GENERAL TASK A10: Routine measures at terminal / measures in response to COVID-19 event**			
**Task no.**	**Specific task description**	**Responsible agency** (committee, competent authority, co-competent authority/ service provider)	**Liaison persons** 1. Main contact 2. Alternate contact
**A10.1.**	**Ensure compliance with personal protective measures at terminal**<br>• Face mask rules<br>• Hand hygiene<br>• Physical distancing (passenger lanes, floor markers)<br>• Different flows	• ………………………	1. …….. 2. ……..
**A10.2.**	**Monitor and replace:**	• ………………………	1. …….. 2. ……..

	• EU certified PPE regularly (e.g. 3 sets of PPE per cruise ship call per staff)<br>• hand hygiene items (soap, alcohol-based hand rub stations) around port facilities<br>• Supplies in isolation area		
**A10.3.**	**Ensure hand washing stations or alcohol-based hand rub stations placed in prominent areas of port facility (e.g. check-in/entrances)**	• ………………………	1. ……..<br>2. ………
**A10.4.**	**Work schedule management at port (work and break schedule review and adjustment to avoid overlap)**	• ………………………	1. ……..<br>2. ………
**A10.5.**	**Health screening of terminal staff for entry to port/terminal (same screening protocols as travellers)**	• ………………………	1. ……..<br>2. ………
**A10.6.**	**Diagnostic testing of terminal workers (if conducted)**	• ………………………	1. ……..<br>2. ………
**A10.7.**	**Develop, update and display health promotion material**	• ………………………	1. ……..<br>2. ………
**A10.8.**	**Ventilation management terminal stations**	• ………………………	1. ……..<br>2. ………
**A10.9.**	**WC management at terminal stations**	• ………………………	1. ……..<br>2. ………
**A10.10.**	**Ensuring cleaning and disinfection of isolation area after each use**	• ………………………	1. ……..<br>2. ………
**A10.11.**	**Ensuring appropriate ventilation of isolation area**	• ………………………	1. ……..<br>2. ………

**A10.12.**	**Transport of possible/confirmed case to temporary isolation area at port**	• ………………………	1. ……..<br>2. ………
**A10.13.**	**Care of possible/confirmed case at temporary isolation area at port**	• ………………………	1. ……..<br>2. ………
**A10.14.**	**Record-keeping of personnel working in isolation area at port**	• ………………………	1. ……..<br>2. ………
**A10.15.**	**Decision about exit/entry screening measures for passengers at ports**	• ………………………	1. ……..<br>2. ………
**A10.16.**	**Conduct of primary health screening measures for passengers at terminal (e.g. visual inspection, collection of health questionnaire and temperature measurement)**	• ………………………	1. ……..<br>2. ………
**A10.17.**	**Conduct secondary screening of travellers (in depth interview, medical examination and laboratory examination, second temperature measurement etc.)**	• ………………………	1. ……..<br>2. ………
**A10.18.**	**Decision to restrict/refuse entry or exit of travellers at port**	• ………………………	1. ……..<br>2. ………
**A10.19.**	**Arrange for contact tracing of affected persons if possible case identified at port terminal**	• ………………………	1. ……..<br>2. ……..
**A10.20.**	**Cleaning and disinfection of terminal facilities before and after each embarkation and ship visit**	• ………………………	1. ……..<br>2. ……..
**A10.21.**	**Special cleaning and disinfection protocols of terminal facilities after possible/confirmed case identified at terminal or if used terminal/port facilities**	• ………………………	1. ……..<br>2. ……..

**A10.22.**	**Cleaning and disinfection of means of transport (e.g. buses, cars etc.) regularly and disinfection when in contact with possible/confirmed case (if different than above)**	• ……………………….	1. ……..<br>2. ……..
**A10.23.**	**Infectious waste management at port facilities**	• ……………………….	1. ……..<br>2. ……..
**A10.24.**	**Luggage disinfection of possible/confirmed cases**	• ……………………….	1. ……..<br>2. ……..
**A10.25.**	**Luggage tracking and management system for possible/confirmed cases**	• ……………………….	1. ……..<br>2. ……..
**A10.26.**	**Disinfection of transport carts/cages after boarding procedure to/from ship**	• ……………………….	1. ……..<br>2. ……..
**A10.27.**	**Management of groups for shore-based excursions and facilitating compliance with physical distancing and separation from other travel groups, verifying PPE available and determining parking spaces for tour buses**	• ……………………….	1. ……..<br>2. ……..

B. RESPONSE PHASE

1B. Tasks for management of COVID-19 event at port

GENERAL TASK B1: COVID-19 public health emergency contingency plan

Task no.	Specific task description	Responsible agency<br>(committee, competent authority, co-competent authority/ service provider)	Liaison persons<br>1. Main contact<br>2. Alternate contact
**B1.1.**	**Activation of the port COVID-19 PHECP**	• ……………………….	1. ……..<br>2. ……..

**B1.2.**	**Deactivation of the port COVID-19 PHECP**	• ………………………	1. ……..<br>2. ……..

GENERAL TASK B2: Detection of COVID-19 event

Task no.	Specific task description	Responsible agency<br>(committee, competent authority, co-competent authority/ service provider)	Liaison persons<br>1. Main contact<br>2. Alternate contact
**B2.1.**	**Receive Maritime Declaration of Health (MDH)**	• ………………………	1. ……..<br>2. ……..
**B2.2.**	**Notification from previous port of call**	• ………………………	1. ……..<br>2. ……..
**B2.3.**	**Notification through National Focal Point**	• ………………………	1. ……..<br>2. ……..
**B2.4.**	**Notification during inspection**	• ………………………	1. ……..<br>2. ……..
**B2.5.**	**Notification at port facilities (e.g. pre-board screening)**	• ………………………	1. ……..<br>2. ……..

GENERAL TASK B3: COVID-19 event verification and preliminary risk assessment

Task no.	Specific task description	Responsible agency<br>(committee, competent authority, co-competent authority/ service provider)	Liaison persons<br>1. Main contact<br>2. Alternate contact
**B3.1.**	**Verification of COVID-19 event**<br>(e.g. collecting further information from ship agent/master/ other authority)	• ………………………	1. ……..<br>2. ……..

**B3.2.**	**Preliminary risk assessment once notified of COVID-19 event**	• ………………………	1. ……..<br>2. ……..
**B3.3.**	**Initial communication to other authorities once COVID-19 event notified on board ship or at port**	• ………………………	1. ……..<br>2. ……..
**GENERAL TASK B4: Cruise ship authorization/free pratique**			
**Task no.**	**Specific task description**	**Responsible agency**<br>**(committee, competent authority, co-competent authority/ service provider)**	**Liaison persons**<br>**1. Main contact**<br>**2. Alternate contact**
**B.4.1.**	**Allow or refuse entry of ship (cruise ship authorization procedure)**	• ………………………	1. ……..<br>2. ……..
**B4.2.**	**Permit free pratique with on-site visit including medical personnel**	• ………………………	1. ……..<br>2. ……..
**B4.3.**	**Determine docking area for the affected ships**	• ………………………	1. ……..<br>2. ……..
**B4.4.**	**Provide floating vessel (in context of free pratique) for transport of medical personnel**	• ………………………	1. ……..<br>2. ……..
**B4.5.**	**Provision of customs documents, emergency materials and supplies for ship if needed**	• ………………………	1. ……..<br>2. ……..
**GENERAL TASK B5: Health measures implementation**			
**Task no.**	**Specific task description**	**Responsible agency**<br>**(committee, competent authority, co-competent authority/ service provider)**	**Liaison persons**<br>**1. Main contact**<br>**2. Alternate contact**
**B5.1.**	**Conduct risk assessment of public health event at port level**		

**B5.2.**	**Decision for health measures implementation**	• ………………………	1. ……..<br>2. ……..
**B5.3.**	**Send epidemiological data of the ship from local to central level**	• ………………………	1. ……..<br>2. ……..
**B5.4.**	**Assessment of case to decide if requires hospitalization ashore or can be transferred to isolation facility ashore**	• ………………………	1. ……..<br>2. ……..
**B5.5.**	**Arrange medical evacuation from ship if needed**	• ………………………	1. ……..<br>2. ……..
**B5.6.**	**Arrange for immediate isolation and testing of possible case**	• ………………………	1. ……..<br>2. ……..
**B5.7.**	**Verification that passenger/crew locator form (PLF) completed by all pax. and crew on board ship**	• ………………………	1. ……..<br>2. ……..
**B5.8.**	**Collect information about contacts of possible/confirmed case (on board ship or ashore)**	• ………………………	1. ……..<br>2. ……..
**B5.9.**	**Disembarkation of confirmed COVID-19 case**	• ………………………	1. ……..<br>2. ……..
**B5.10.**	**Disembarkation of close contacts**	• ………………………	1. ……..<br>2. ……..
**B5.11.**	**Determine which hospitals/medical facilities in local area receive patients and inform medical facility/hospital of arrival of COVID-19 case**	• ………………………	1. ……..<br>2. ……..

**B5.12.**	**Inform ashore designated isolation/quarantine facility about arrival COVID-19 case**	• ………………………	1. ……..<br>2. ……..
**B5.13.**	**Transport of COVID-19 case to hospital if required**	• ………………………	1. ……..<br>2. ……..
**B5.14.**	**Arrange interpreters**	• ………………………	1. ……..<br>2. ……..
**B5.15.**	**Transport of COVID-19 case (e.g. asymptomatic or mildly symptomatic) to isolation facility**	• ………………………	1. ……..<br>2. ……..
**B5.16.**	**Health monitoring of cases in isolation facilities**	• ………………………	1. ……..<br>2. ……..
**B5.17.**	**Registration of COVID-19 case in national web-based system**	• ………………………	1. ……..<br>2. ……..
**B5.18.**	**Disembarkation of close contacts**	• ………………………	1. ……..<br>2. ……..
**B5.19.**	**Support for persons in isolation (e.g. providing food, medications etc.)**	• ………………………	1. ……..<br>2. ……..
**B5.20.**	**Decision to release cases from isolation facilities**	• ………………………	1. ……..<br>2. ……..
**B5.21.**	**Transport of close contacts to designated quarantine facility**	• ………………………	1. ……..<br>2. ……..

**B5.22.**	**Registration of close contacts in national web-based system**	• ………………………	1. ……..<br>2. ……..
**B5.23.**	**Active health monitoring of close contacts ashore**	• ………………………	1. ……..<br>2. ……..
**B5.24.**	**Support for persons in isolation (e.g. providing food, medications etc.)**	• ………………………	1. ……..<br>2. ……..
**B5.25.**	**Psychological support for people in quarantine and isolation if needed**	• ………………………	1. ……..<br>2. ……..
**B5.26.**	**Arrangement of early testing for all symptomatic and asymptomatic close contacts of confirmed case**	• ………………………	1. ……..<br>2. ……..
**B5.27.**	**Conduct of contact tracing if needed**	• ………………………	1. ……..<br>2. ……..
**B5.28.**	**Arrange of repatriation of travellers**	• ………………………	1. ……..<br>2. ……..
**B5.29.**	**Conduct ship inspection if confirmed case identified on board**	• ………………………	1. ……..<br>2. ……..
**B5.30.**	**Supervision and implementation of health measures implemented on ship**<br>• Cleaning and disinfection of contaminated areas/objects<br>• Waste management / disposal<br>• Replace air filters<br>• Preventive measures for next voyage (e.g. active surveillance)<br>• Any other health measures required	• ………………………	1. ……..<br>2. ……..

**B5.31.**	**Review proof of measures and take decision if satisfied with health measures implemented on board ship, note measures in Ship Sanitation Certificate**	• ………………………	1. ……..<br>2. ……..
**B5.32.**	**At departure inform competent authority at next known port of call about evidence found on ship and control measures implemented/required**	• ………………………	1. ……..<br>2. ……..
**B5.33.**	**Treatment of baggage, cargo, containers, conveyances, goods, postal parcels or human remains to remove infection if needed**	• ………………………	1. ……..<br>2. ……..
**B5.34.**	**Safe handling and transport of human remains**	• ………………………	1. ……..<br>2. ……..
**B5.35.**	**Isolation and quarantine of ships when public health risk on board**	• ………………………	1. ……..<br>2. ……..
**B5.36.**	**Seizure / destruction of infected ship and other inanimate objects**	• ………………………	1. ……..<br>2. ……..
**B5.37.**	**Supervision of and removal of safe disposal of contaminated matter from ship**	• ………………………	1. ……..<br>2. ……..
**B5.38.**	**Interview possible case and obtain medical history and detailed travel history of possible case and close contacts if possible case detected at port during pre-board screening**	• ………………………	1. ……..<br>2. ……..
**B5.39.**	**Review proof of medical examination or microbiological diagnostic test as needed**	• ………………………	1. ……..<br>2. ……..
**B5.40.**	**Review proof of vaccination as needed**	• ………………………	1. ……..<br>2. ……..

B. RESPONSE PHASE

2B. Channels of communication during COVID-19 event management

GENERAL TASK B6: Internal communications			
Task no.	Specific task description	Responsible agency (committee, competent authority, co-competent authority/ service provider)	Liaison persons 1. Main contact 2. Alternate contact
B6.1.	Internal communication within port	• ………………………	1. ……..<br>2. ……..
B6.2.	Communication with IHR national focal point (NFP)	• ………………………	1. ……..<br>2. ……..
B6.3.	Communicate with media about public health event (public information/media management)	• ………………………	1. ……..<br>2. ……..
B6.4.	Communicate to alternate port and ask to manage public health event if capacities unavailable	• ………………………	1. ……..<br>2. ……..
