# Supplementary material for: Re-Starting the Cruise Sector during the COVID-19 Pandemic in Greece: Assessing Effectiveness of Port Contingency Planning
Source: Int J Environ Res Public Health. 2022 Oct 14;19(20):13262. doi: 10.3390/ijerph192013262 (PMC9603745; doi:10.3390/ijerph192013262)
Supplement: Supplementary file 1 [file ijerph-19-13262-s001.zip › TableS3_IAR_Discussion_Templates.pdf]

IN-ACTION REVIEW: ANALYSIS OF COVID-19 EVENTS – Facilitated discussion templates

SESSION 1: CRUISE SHIP A			
Duration:	35 minutes	Time:	9:40-10:15
Method of review:	Root-Cause Analysis		

Root-Cause Analysis Questions:	
A1	*At first look what was the problem? How do we define the problem?*
A2	*What was the impact of the problem? What did it cause?*
B1	*What caused the problem described from A1?*
C1	*What was the cause of the reasons listed in B1?*
D1	*What are possible solutions to prevent the root causes from C1?*
E1	*Is there anything you think could have been done differently from your authority?*

A1. Problem:

- ……………………………………….
- ……………………………………….

A2. Impacts:

- ……………………………………….
- ……………………………………….

**B1. Why did this happen:**	……………	……………	……………	……………
**C1. Root Cause:**	……………	……………	……………	……………
**D1. Possible actions:**	……………	……………	……………	……………

E1. Lookback	
Port authority representative:	
Local Health Authority representative:	
Local Port Agency representative:	
Coast guard representative:	

SESSION 2: CRUISE SHIP B		
35 minutes	Time:	10:15-10:50
Analyse challenges and good practices		

	CHALLENGE 1:
Example	• ....<br>• ....
Impact	• ....<br>• ....
Limiting conditions	• ....<br>• ....
Actions	• ....<br>• ....

	GOOD PRACTICE 1:
Example	• ....<br>• ....
Impact	• ....<br>• ....
Enabling conditions	• ....<br>• ....
Action needed to maintain good practice?	• ....<br>• ....

SESSION 3: CRUISE SHIP C			
Duration:	20 minutes	Time:	10:50-11:10
Method of review:	Root-Cause Analysis		

Root-Cause Analysis Questions:	
A1	*At first look what was the problem? How do we define the problem?*
A2	*What was the impact of the problem? What did it cause?*
B1	*What caused the problem described from A1?*
C1	*What was the cause of the reasons listed in B1?*
D1	*What are possible solutions to prevent the root causes from C1?*
E1	*Is there anything you think could have been done differently from your authority?*

A1. Problem:

- …………………………………………
- …………………………………………

A2. Impacts:

- …………………………………………
- …………………………………………

**B1. Why did this happen:**	……………	……………	……………	……………
**C1. Root Cause:**	……………	……………	……………	……………
**D1. Possible actions:**	……………	……………	……………	……………

**E1. Lookback**	
Port authority representative:	
Local Health Authority representative:	
Local Port Agency representative:	
Coast guard representative:	

SESSION 4: Review of good practices for COVID-19 event management			
Duration:	15 minutes	Time:	11:10-11:25
Method of review:	Facilitated Look Back		

**Description of event:**	*Describe if possible an example of COVID-19 event at port that went well, that went according to plan etc.*
**What went well:**	*During management of the event, what went well? What were good practices that were implemented?*
**Conditions for good practices:**	*How was the management able to go well? What facilitates the good practice?*
**How to sustain:**	*What is required for the good practice to be maintained?*

SESSION 5: URGENT ISSUES AND CHALLENGES FORESEEN FOR 2022			
Duration:	25 minutes	Time:	11:25-11:50
Method of review:	Group discussion		

Current challenges the port is facing or foresee they may face in ongoing COVID-19 response:

**Challenge:**	• ……………………………………….<br>• ………………………………………
**Concrete examples if available:**	• ……………………………………….<br>• ………………………………………
**Discussion about possible solutions:**	• ……………………………………….<br>• ………………………………………
