# Supplementary figures and images for: Re-Starting the Cruise Sector during the COVID-19 Pandemic in Greece: Assessing Effectiveness of Port Contingency Planning
Source: Int J Environ Res Public Health. 2022 Oct 14;19(20):13262. doi: 10.3390/ijerph192013262 (PMC9603745; doi:10.3390/ijerph192013262)

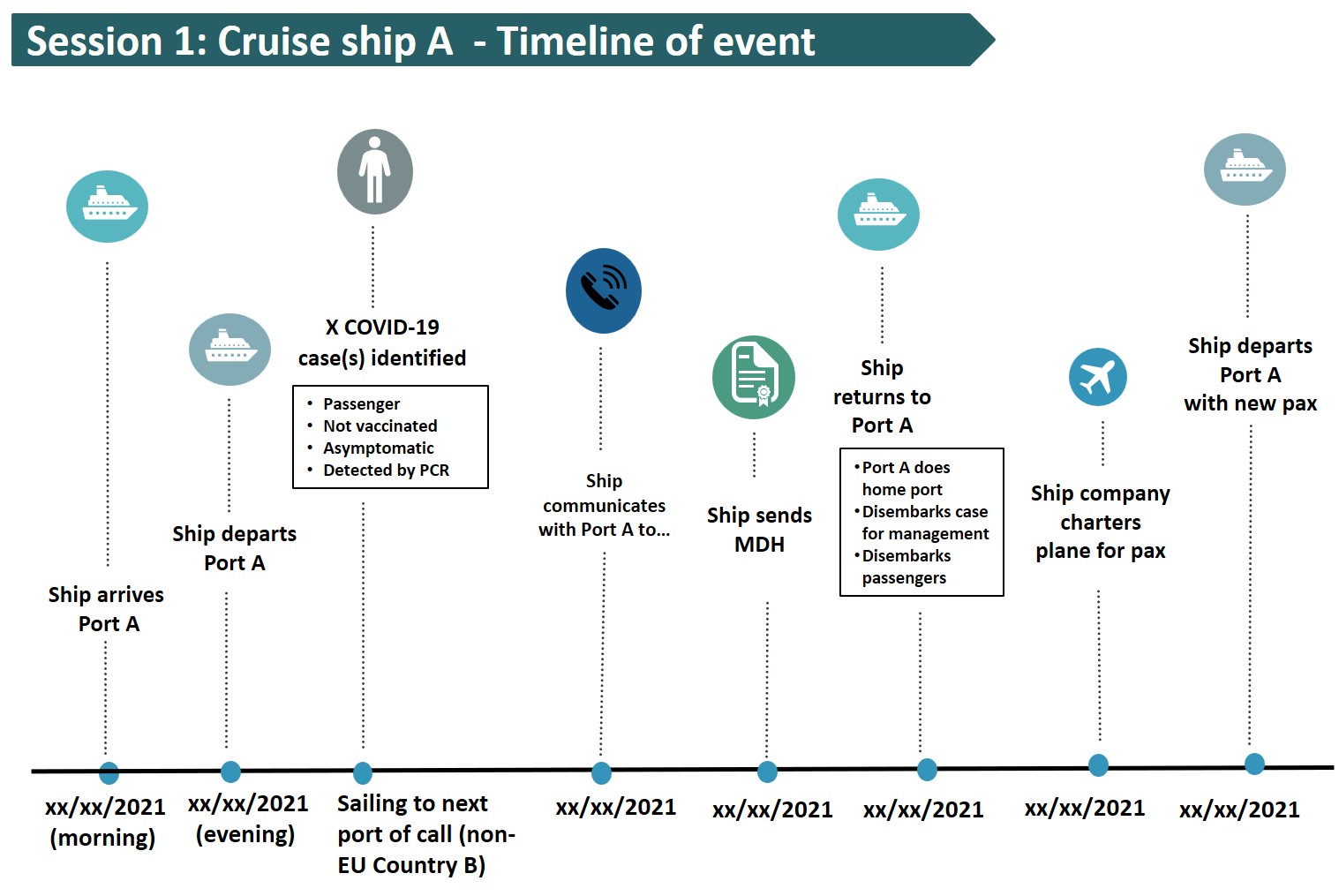

Supplement: Supplementary file 1 [file ijerph-19-13262-s001.zip › FigureS1_IAR_COVID_Timeline.jpg]
